# Elevated Levels of Plasma Phosphorylated Tau 181 (pTau181) Associated With Opioid Use to Guide Medication Titration Over a Clinically Relevant Short Timescale

**DOI:** 10.7759/cureus.79268

**Published:** 2025-02-19

**Authors:** Emily J Hanson, Karl F Berner, Jon Berner

**Affiliations:** 1 Biology, Woodinville Psychiatric Associates, Woodinville, USA; 2 Research, Woodinville Psychiatric Associates, Woodinville, USA; 3 Psychiatry, Woodinville Psychiatric Associates, Woodinville, USA

**Keywords:** amyloid-tau-neurodegeneration profile, lithium, neurodegeneration, opioids, pain, rapamycin

## Abstract

Background: The identification of many medications that delay neurodegeneration in animal models has created too many combinations to try in patients when time is short. We hypothesized that biomarkers of premature neuronal aging that are part of the amyloid-tau-neurodegeneration (ATN) profile, namely amyloid-β ratio, phosphorylated tau 181 (pTau181), and neurofilament light chain (NfL), could provide tools to optimize treatment in single-patient trials rapidly.

Methods: We retrospectively analyzed these biomarkers in patients with extensive neuropsychiatric polypharmacy and premature neuronal aging. We investigated whether ATN profile biomarkers were associated with age, gender, metabolic syndrome markers, and medication use. Additionally, two case reports provided examples of ATN biomarker application in clinical settings.

Results: We identified 113 patients with plasma ATN profiles. Of 80 of those patients, clinical phenotypic data were available. Among these 80 patients, pTau181 was elevated in 31 (38.75%), amyloid-β ratio was below normal ranges in 11 (13.75%), and NfL was elevated in three (3.75%). The biomarkers correlated with age, as expected. Opioid use was significantly associated with pTau181 (p = 0.004) and NfL (p = 0.002), also after Bonferroni correction (both p < 0.05), but not with amyloid-β ratio. The biomarkers were not associated with other medication use.

Conclusion: It is now possible to identify the overlaps between complex behavioral phenotypes (pain and cognition), plasma endophenotypes (ATN profile), and medication-targeted components of age-related pathophysiology. The current study provides a proof of concept; future research should focus on single-patient trials in patients with premature neuronal aging, where medication and dosage choices are based on individual ATN profiles. To facilitate such single-patient trials, funding is needed to promote the use of repurposed generic treatments, educate patients and providers regarding optimization principles, and continue developing sensitive biomarkers. Together, these can ensure the rapid progress of single-patient trials for treatment optimization.

## Introduction

Age-related neurodegeneration, such as, for instance, seen in Alzheimer’s disease (AD) and Lewy body dementia, is a complex process, and there is currently no consensus on etiology. To slow age-related neurodegeneration in animal disease models, treatments with combinations of multiple medications are generally the most effective, consistent with the notion that aging is controlled by a complex network of interacting intracellular signaling pathways [1]. For instance, recent data in the model organism *Drosophila melanogaster* showed a substantial synergistic effect of lithium and rapamycin in combination (30% lifespan extension) compared with either compound alone (11% lifespan extension each) [2]. This finding is particularly interesting because these drugs are inexpensive and managed with a reasonable safety profile in diverse human populations.

All ongoing human trials of rapamycin and lithium are limited to evaluating single agents, typically over long treatment times [3,4]. As a result, treatment with a combination of rapamycin and lithium to promote health during aging has not yet been tested in clinical trials. There are various barriers to researching such treatments, including the generic status of these medications without industry financial support, the minimal availability of a clinical labor force with intersectional training, and perceived regulatory monitoring compliance costs. Until recently, an additional constraint in this field was the difficulty in cost-effectively gathering tissue data with a time constant of response, allowing for multiple medication adjustments within the lifespan of a patient. That is, analyzing cerebrospinal fluid (CSF) is invasive and expensive, detecting changes in magnetic resonance imaging (MRI) volume is slow and has small effect sizes, and performing positron-emission tomography (PET) scans is expensive while access is geographically limited [5].

The recent commercial introduction of tests for plasma biomarkers of neurodegeneration allows for more rapid optimization of prophylactic treatments against neurodegeneration. The amyloid-tau-neurodegeneration (ATN) profile is determined in plasma based on the biomarkers amyloid-β ratio, phosphorylated tau 181 (pTau181), and neurofilament light chain (NfL). It is used to evaluate age-related mild cognitive decline. The presence of amyloid-β plaques and intracellular tau aggregates in the brain is a hallmark of several neurodegenerative diseases, and plasma NfL is a nonspecific marker of neuronal injury [6,7]. Extensive research has shown the relevance of the ATN profile in CSF for diagnosing AD and AD-related dementias [7]. The plasma ATN profile may allow for multiple treatment trials in an individual patient over a span of one to five years, a time span more congruent with clinical needs associated with neurodegeneration prophylaxis.

This retrospective analysis of plasma ATN profiles aimed to summarize clinical experience with them, as they were previously studied only in CSF. Our first objective was to assess whether medication use or standard laboratory data were associated with any of the biomarkers of the ATN profile. The second objective was to describe the implications of the ATN profile in two case reports of patients with premature neuronal aging who were in treatment with lithium or rapamycin.

This article was previously posted to the bioRxiv preprint server on December 4, 2024 (doi: 10.1101/2024.12.01.626163).

## Materials and methods

Study population

The study included patients who presented at a psychiatric practice with subjective cognitive complaints and/or reasonable concerns of premature neuronal aging risk for whom, in the course of normal management, ATN profiles were obtained. Data from this retrospective study were obtained between February 17, 2024, and July 3, 2024.

Clinical data were collected through chart review, including demographic details, laboratory results, whether the patient had tried more than one antidepressant, and which psychiatric medications (i.e., lithium, benzodiazepine, antipsychotic, anticonvulsant, opioid, ketamine, psychostimulant) the patient was taking during their ATN testing. Given that all chronic patients were seen in the course of routine practice, it was highly likely that the current medication list allowed inferences regarding psychiatric comorbidity in each individual patient.

Informed consent

This retrospective chart analysis of diagnostic test results from patients treated with Food and Drug Administration (FDA)-approved medications as part of standard clinical practice was exempt from Institutional Review Board approval under Category 2 of the Basic Health and Human Services Policy for Protection of Human Research Subjects Subpart A Section 46.101 [8]. No informed consent was required.

ATN profile biomarker data

As part of diagnostic testing, ATN profile biomarkers had previously been measured. In short, LabCorp (Burlington, NC) collected and analyzed plasma samples. Concentrations of amyloid-β 42 (Aβ42) and amyloid-β 40 (Aβ40) were measured with a chemiluminescence enzyme immunoassay based on Sysmex reagents and technology (Sysmex, Kobe, Japan). The amyloid-β ratio (Aβ42/Aβ40) was subsequently calculated. An amyloid-β ratio of >0.102 was considered normal. Concentrations of pTau181 and NfL were analyzed using electrochemiluminescence immunoassays based on Roche reagents and technology (Roche Diagnostics, Indianapolis, IN). The normal pTau181 range for individuals aged 20-55 years is 0.00-0.95 pg/mL, while the normal range for individuals over 55 years is 0.00-0.97 pg/mL. The normal NfL range is 0.00-2.13 pg/mL.

Statistical analysis

Patient data were entered into an Excel database (Excel version 2412, Microsoft, Redmond, WA) and exported into GNU PSPP version 2.0.1-g486e58 (https://www.gnu.org/software/pspp/) for data analysis. Variables are presented as mean with standard deviation (SD), median, range, or proportion. Pearson correlation coefficients (r) were calculated between ATN variables and clinical phenotypes. Bonferroni adjustment was performed post hoc (for eight comparisons) on two-tailed statistical findings related to current medication use. A two-tailed unpaired t-test was used to identify significant differences between the groups. A p value of <0.05 was considered significant. All graphs were generated in GraphPad Prism version 10.4.0 (Dotmatics, Boston, MA).

## Results

Patients

In total, 113 patients with premature neuronal aging were identified through chart reviews. The mean age of these patients was 60.4 (SD = ±12.3) years, and the median age was 61 years. Most of these were female patients (n = 77/113, 68%). The patients had a mean body mass index (BMI) of 28.4 (SD = ±6.3) kg/m^2^. Clinical data are presented in Table 1. Fee for service insurance and geographic location implied that the patients were in the upper 75% of the United States socioeconomic distribution. Of these patients, 36 had tried >1 antidepressant. At the time of the blood draw for ATN testing, the medications they were using were lithium (n = 12), benzodiazepine (n = 61), antipsychotic (n = 36), anticonvulsant (n = 53), opioid (n = 18), ketamine (n = 22), and psychostimulant (n = 45). All patients were psychiatrically stable, implied by a follow-up frequency between 3 and 18 months.

**Table 1:** Clinical data of patients (n = 113) Not all data were available for all patients: ^a^height and BMI were available for 99 patients, ^b^weight for 103 patients, ^c^FBS for 49 patients, ^d^TG/HDL ratio for 46 patients, ^e^insulin-to-BMI ratio and RDW for 35 patients, and ^f^neutrophil-to-lymphocyte ratio for 32 patients BMI, body mass index; FBS, fasting blood sugar; RDW, red cell distribution width; SD, standard deviation; TG/HDL, triglyceride-to-high-density lipoprotein ratio

Clinical variable	Mean (±SD)	Median	Minimum	Maximum	Range
Age (years)	60.4 (±12.3)	61	23	92	69
Height^a^ (cm)	168.9 (±10.5)	167.6	142.6	198.1	55.9
Weight^b^ (kg)	81.1 (±20.3)	79.8	44.9	162.8	117.9
BMI^a^ (kg/m^2^)	28.4 (±6.3)	27.2	17.6	50.1	32.4
FBS^c^ (mg/dL)	97.9 (±19.7)	95	67	164	97
TG/HDL ratio^d^	2.8 (±2.3)	1.9	0.5	11	10.4
Insulin-to-BMI ratio^e^	2.1 (±1.4)	1.5	0.1	6	6
RDW^e^ (%)	13.2 (±1.7)	12.9	11.7	21.1	9.4
Neutrophil-to-lymphocyte ratio^f^	2.1 (±0.9)	1.9	0.8	4.4	3.5

ATN profile biomarkers were correlated with age

**Figure 1:**
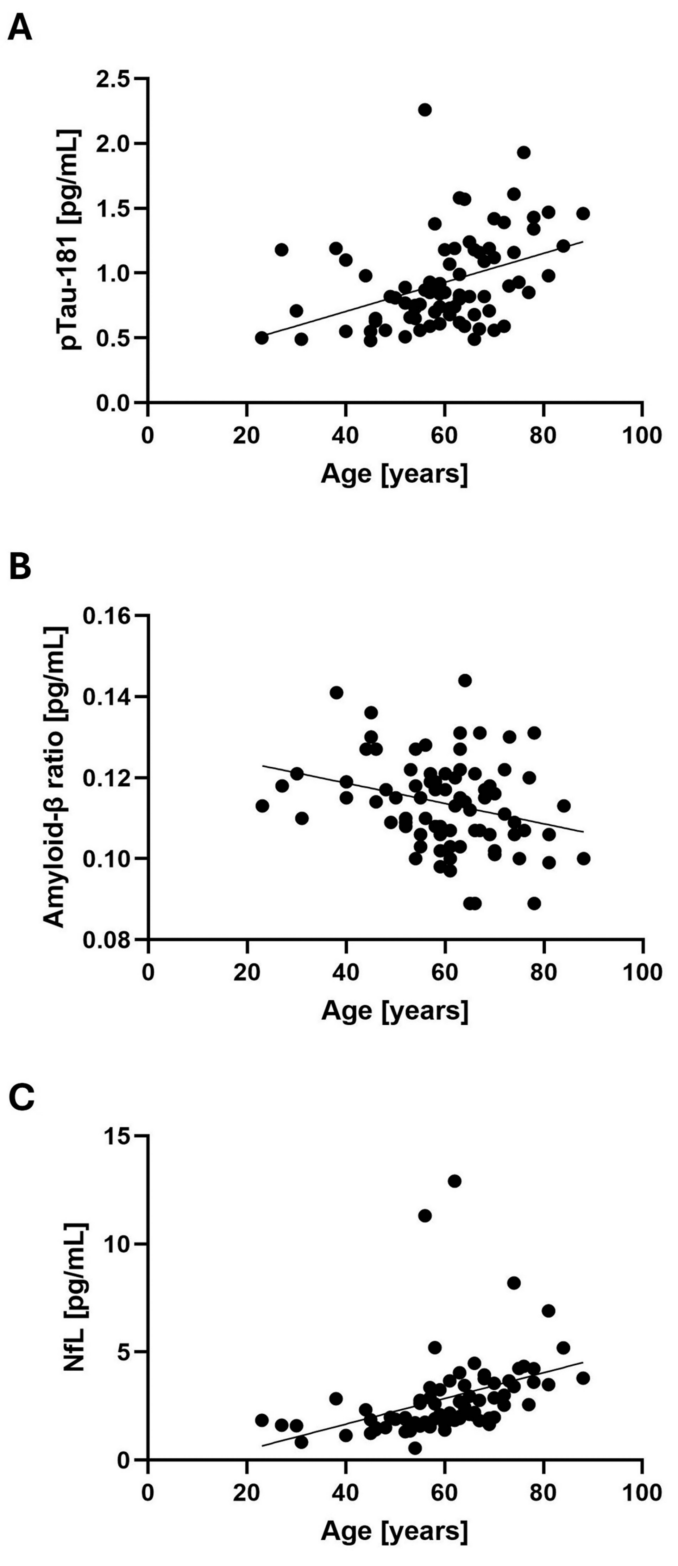
Correlations between ATN profile biomarkers and age Scatter plots of the ATN profile biomarkers and age of the patients (n = 80). Each black dot represents a single value in one patient, and the thin line represents the simple linear regression that was determined with a Pearson correlation test. (A) Correlation between pTau181 concentration and age (r = 0.405, p < 0.000). (B) Correlation between NfL concentration and age (r = 0.384, p < 0.000). (C) Correlation between amyloid-β ratio and age (r = -0.285, p < 0.010) ATN, amyloid-tau-neurodegeneration; NfL, neurofilament light chain; pTau181, phosphorylated tau 181

Full phenotypic profiles and plasma ATN profiles were available for 80 patients. The mean pTau181 concentration was 0.93 (SD = ±0.35) pg/mL, the mean NfL concentration was 2.85 (SD = ±1.97) pg/mL, and the mean amyloid-β ratio was 0.1 (SD = ±0.01) (Table 2). Of these 80 patients, 31 (38.75%) had a pTau181 concentration above normal ranges, 11 (13.75%) had an amyloid-β ratio below normal ranges, and three (3.75%) had an NfL concentration above normal ranges. Sixty-nine patients (86.25%) had an amyloid-β ratio within normal ranges, while 11 patients (13.75%) had an amyloid-β ratio below normal ranges. Seventy-seven patients (96.25%) had an NfL within normal ranges, and three patients (3.75%) had an NfL above normal ranges. None of the 80 patients tested had values of all three biomarkers in abnormal ranges. Age was found to be correlated with pTau181 concentration (r = 0.405, p < 0.000), NfL concentration (r = 0.384, p < 0.000), and amyloid-β ratio (r = -0.285, p < 0.010) (Figure 1).

**Table 2:** ATN profiles in patients (n = 80) Aβ40, β-amyloid 40; Aβ42, β-amyloid 42; ATN, amyloid-tau-neurodegeneration; NfL, neurofilament light chain; pTau181, phosphorylated tau 181; SD, standard deviation

ATN profile biomarker	Mean (±SD)	Range
pTau181 (pg/mL)	0.93 (±0.35)	0.48-2.26
pTau181 within normal ranges	0.71 (±0.13)	0.45-0.93
pTau181 above normal ranges	1.3 (±0.28)	0.98-2.26
Aβ42 (pg/mL)	19.9 (±4.4)	9.7-35.8
Aβ40 (pg/mL)	175.5 (±33.2)	91.5-188.9
Amyloid-β ratio	0.1 (±0.01)	0.09-0.14
Amyloid-β ratio within normal ranges	0.12 (±0.01)	0.10-0.14
Amyloid-β ratio below normal ranges	0.096 (±0.005)	0.089-0.10
NfL (pg/mL)	2.85 (±1.97)	0.56-12.9
NfL within normal ranges	2.54 (±1.14)	0.56-6.9
NfL above normal ranges	10.79 (±2.39)	8.19-12.9

ATN profile biomarkers were not correlated with metabolic syndrome markers

**Figure 2:**
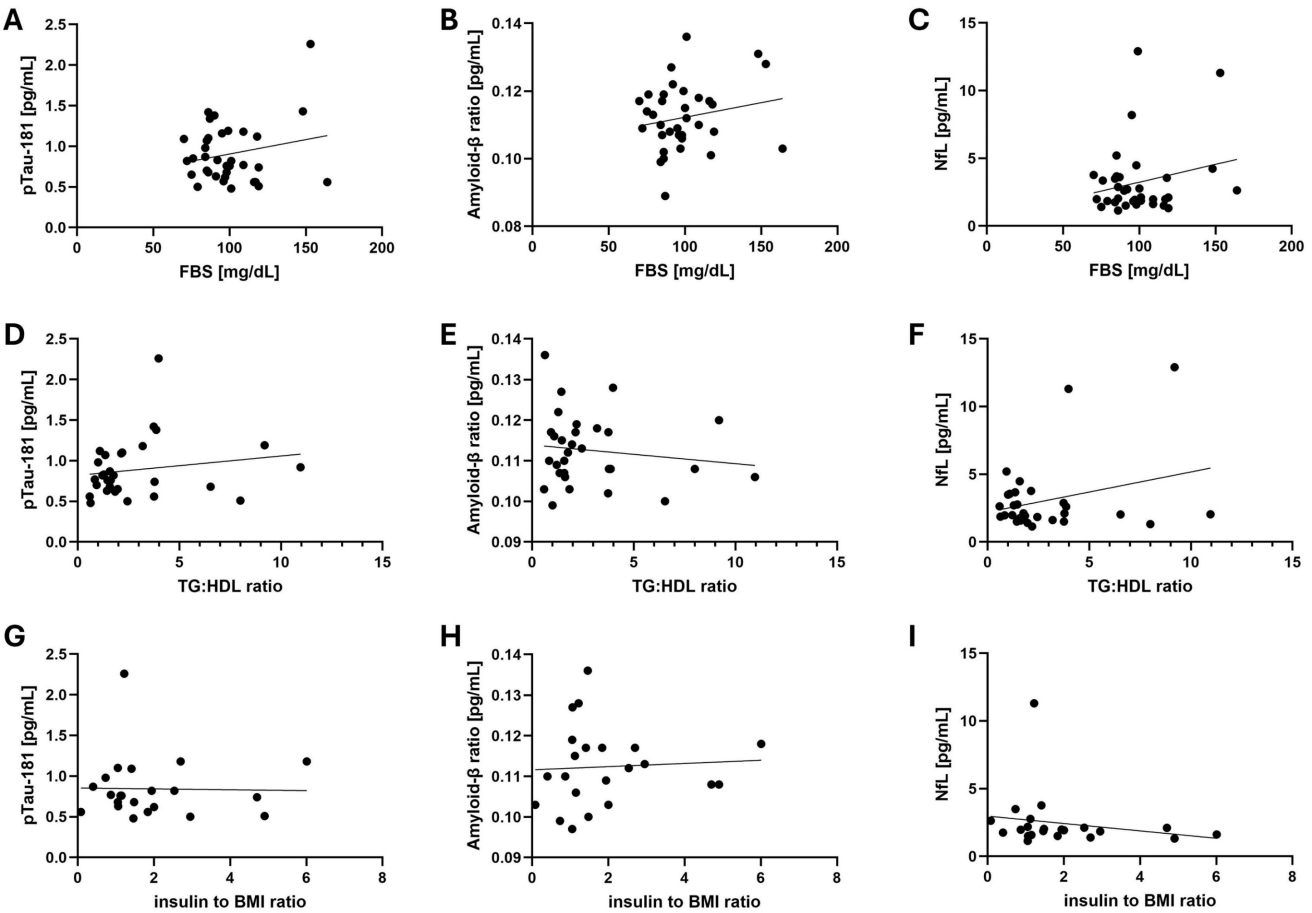
No correlation between ATN profile biomarkers and metabolic syndrome markers Scatter plots of the ATN profile biomarkers vs. the metabolic syndrome markers in the 80 patients. (A) pTau181 concentration vs. FBS (r = 0.211). (B) Amyloid-β ratio vs. FBS (r = 0.194). (C) NfL concentration vs. FBS (r = 0.221). (D) pTau181 concentration vs. TG/HDL ratio (r = 0.170). (E) Amyloid-β ratio vs. TG/HDL ratio (r = -0.139). (F) NfL concentration vs. TG/HDL ratio (r = 0.290). (G) pTau181 concentration vs. insulin-to-BMI ratio (r = -0.050). (H) Amyloid-β ratio vs. insulin-to-BMI ratio (r = 0.060). (I) NfL concentration vs. insulin-to-BMI ratio (r = -0.122) Each black dot represents a single value in one patient, and the thin line represents the simple linear regression that was determined with a Pearson correlation test. FBS was available for 35 patients, TG/HDL ratio for 30 patients, and insulin-to-BMI ratio for 22 patients ATN, amyloid-tau-neurodegeneration; BMI, body mass index; FBS, fasting blood glucose; NfL, neurofilament light chain; pTau181, phosphorylated tau 181; TG:HDL, triglyceride to high-density lipoprotein

Since metabolic syndrome markers like fasting blood sugar, triglyceride-to-high-density lipoprotein ratio, and insulin-to-BMI ratio were not available for all 80 patients, the potential association with biomarkers of the ATN profile was analyzed in smaller subsets. ATN profile biomarkers were not associated with any of the metabolic syndrome markers in the subset of patients with a full metabolic profile (Figure 2).

Opioid use was associated with pTau181 and NfL

**Figure 3:**
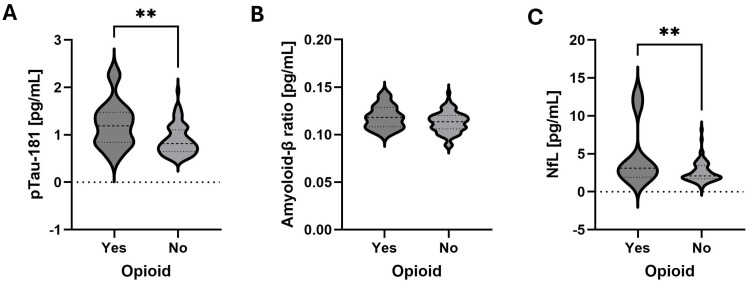
Opioid use associated with higher pTau181 and NfL Violin plots of ATN profile biomarkers in 80 patients, 10 of which were using opioids at the time. A two-tailed unpaired t-test was used to identify significant differences between the groups. The dashed line in the violin plot indicates the median, and the dotted lines indicate the quartiles. (A) pTau181 concentration. (B) NfL concentration. (C) amyloid-β ratio ^**^p< 0.005 ATN, amyloid-tau-neurodegeneration; NfL, neurofilament light chain; pTau181, phosphorylated tau 181

We assessed associations between current medication use or previously failed medication(s) and the biomarkers of the ATN profile. We found that opioid use was significantly associated with pTau181 concentration (r = 0.315, p = 0.004) (Figure 3A) and NfL concentration (r = 0.341, p = 0.002) (Figure 3B) but not with the amyloid-β ratio (r = 0.157, p = 0.165) (Figure 3C). After Bonferroni correction for multiple comparisons across eight medication classes, pTau181 and NfL concentrations were still significantly associated with opioid use (p < 0.05).

Psychostimulant use was significantly but weakly associated with the amyloid-β ratio (r = 0.023, p = 0.039). After Bonferroni correction, this finding was no longer significant (p > 0.05). Other medications and previous medication failure(s) were not associated with any of the biomarkers of the ATN profile.

Case report 1: lithium treatment

**Figure 4:**
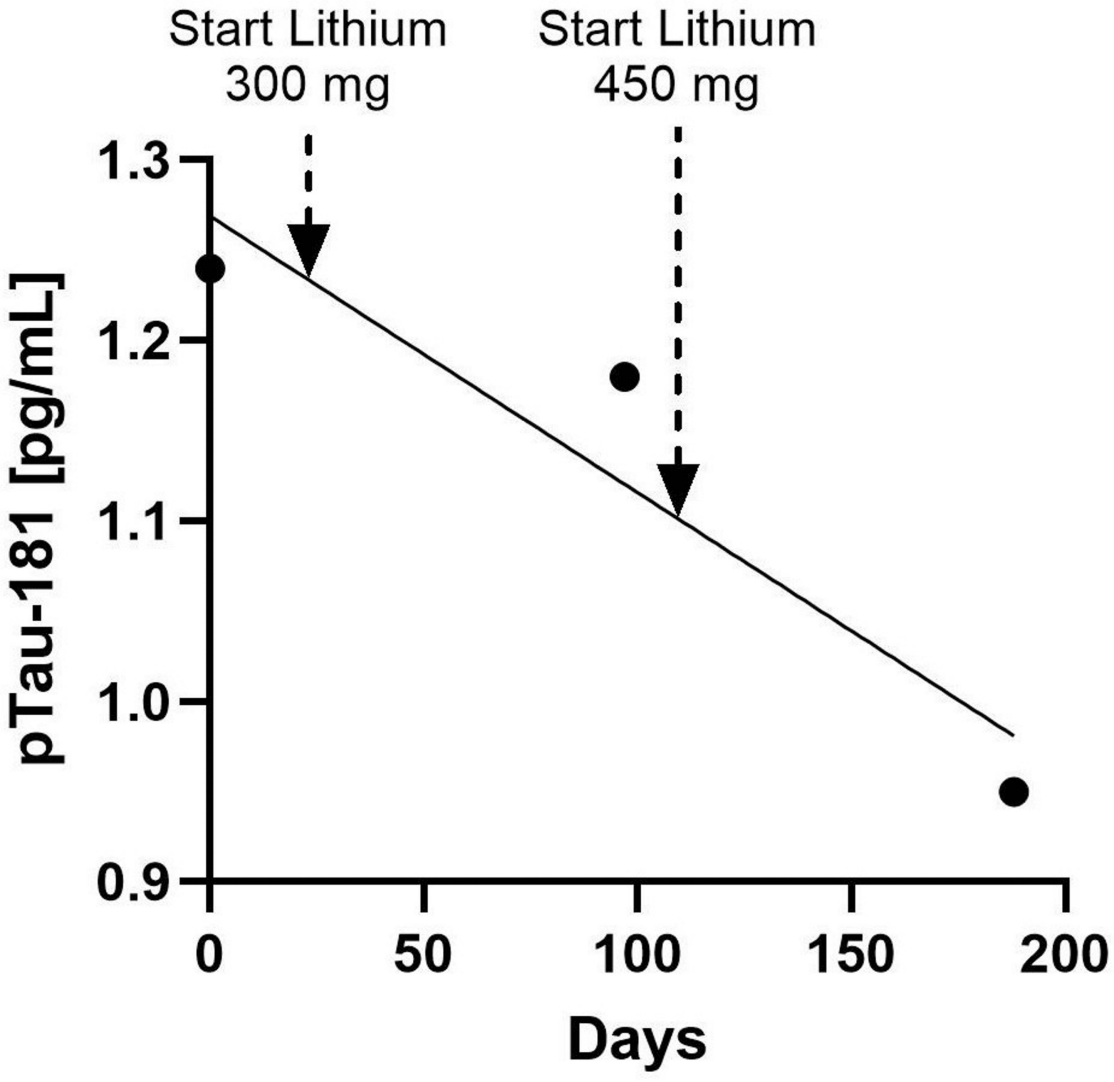
pTau181 concentration during lithium treatment in Case 1 The black dots indicate the concentration on the test days (days 0, 97, and 193). Lithium 300 mg was started on day 23, and lithium 450 mg was started on day 107. The diagonal line indicates the simple linear regression (r= 0.89) pTau181, phosphorylated tau 181

A 65-year-old female patient presented in March 2021, at 61 years old, for bipolar disorder. Her disorder remained stable on lamotrigine 100 mg and Adderall 30 mg until February 2024. At that time, given the progression of irritability and concentration disturbance, in combination with the dense penetrance of Parkinson’s disease in her mother’s line, her baseline ATN profile was assessed. Her pTau181 and NfL concentrations were above the normal range, and her amyloid-β ratio was below the normal range. Based on her ATN profile, the patient elected to start with lithium. Subsequently, over the next six months, her irritability resolved (reported by patient and spouse), and her pTau181 concentrations decreased to within the normal range for her age. Longitudinal data of her pTau181 concentrations are presented in Table 3 and Figure 4.

**Table 3:** ATN profile of case report 1 Aβ40, β-amyloid 40; Aβ42, β-amyloid 42; ATN, amyloid-tau-neurodegeneration; NfL, neurofilament light chain; pTau181, phosphorylated tau 181 ^*^Since day 23 ^**^Since day 107

Day	Lithium dose (mg)	pTau181 (pg/mL)	Aβ42 (pg/mL)	Aβ40 (pg/mL)	Amyloid-β ratio	NfL (pg/mL)
0	0	1.24	14.28	160.73	0.089	2.96
97	300^*^	1.18	17.24	173.79	0.099	3.22
193	450^**^	0.95	12.74	135.11	0.094	2.98

Case report 2: rapamycin prophylaxis

A 63-year-old female patient has been seen in our clinic since 2006. Initially, she presented with a nonspecific mood disorder that was treated with 225 mg venlafaxine and leukoencephalopathy with an associated pain syndrome that was treated with transdermal fentanyl 25 μg/hour. Her subsequent course has been insidiously progressive over 18 years with the emergence of atonic seizures, bipolar disorder, Parkinson's disease, and relentless progression of neurogenic pain. Her mother also has Parkinson’s disease. Numerous evaluations ruled out multiple sclerosis. Multivariate analysis of her CSF metabolome revealed substantial z-decrements in homocarnosine suggestive of accelerated astroglial aging/inflammation; see patient number #895351 in the first figure in [9].

Rapamycin 6 mg weekly was initiated in June 2020 and subsequently titrated to effect over multiple years to 4 mg. During the titration period, she considered suicide due to poor pain control despite 80 mg oxycodone. Her dominant response to rapamycin was near complete resolution of fatigue, which formerly kept her bedbound most of her day. Her secondary response, likely in conjunction with acarbose 300 mg daily (prescribed as prophylaxis for opioid constipation and dementia) and rotation to 16 mg of buprenorphine, was “perfect” pain control. Despite treatment, her pTau181 concentration was 1.07 pg/mL in March 2024, with subsequent longitudinal monitoring planned. Although the patient was subjectively stable with complicated polypharmacy, her laboratory results suggested continued occult central nervous system neurodegeneration. Consistent with the standard of care in other clinical settings (e.g., continued elevated cholesterol after initial statin prophylaxis for secondary prevention of atherosclerosis), prudent management requires discussion of rapamycin dose increase or further augmentation with additional medications.

## Discussion

The chief finding in this retrospective study was that opioid use was significantly associated with elevated concentrations of the neurodegeneration markers pTau181 and NfL in plasma. An additional incidental finding was that high pTau181 had immediate relevance for the clinical management of the antiaging compounds lithium and rapamycin. We included two retrospective case reports to demonstrate the use of ATN profile measurements for optimizing treatment in individual patients.

As the plasma test of ATN profile biomarkers only became commercially available about a year ago, no comparable reports are available in the literature yet. However, the association between opioid use and high plasma pTau181 was consistent with a large study (n = 995) that discovered an association between chronic pain and increased pTau181 in CSF. That study also found an association between chronic pain and total tau (p < 0.04) and between chronic pain and the CSF marker tumor necrosis factor of "M1-like" microglial activation (p < 0.02) [10]. Note that the community study, which included subjects with mild cognitive impairment, subjects with AD, as well as healthy controls, likely sampled an overall less sick population and did not determine axonal degeneration associated with pain as they did not analyze NfL concentration [10]. The preliminary case reports we presented here suggest that lithium and rapamycin may, in select individuals, treat subjective pain complaints and objective markers of central nervous system inflammation.

Chronic lithium use for dementia prophylaxis is massively underutilized in community settings despite double-blind studies documenting efficacy in humans [11] and epidemiological studies linking variable dementia prevalence to trace lithium concentrations in the water supply [12]. Optimal lithium titration for dementia prophylaxis is limited by a host of subtle side effects well known to experienced psychiatric providers [13], strongly arguing against guidelines based on population averages rather than single-patient trials using ATN profiles to guide optimal titration.

Trials of rapamycin for dementia prophylaxis are ongoing [14], and one small randomized, placebo-controlled human trial (n = 115) showed effective prophylaxis against age-related pain in women after 48 weeks on a very low dose of rapamycin (p = 0.02) [15]. Optimal dose titration of rapamycin in the individual patient is even less well-studied than dose titration of lithium, with almost absent structured clinical data regarding side effects between doses typically used for healthy longevity patients (i.e., 1 mg) [16] and the higher doses (i.e., 3-5 mg) used for transplant rejection prophylaxis or systemic lupus erythematosus [17].

We note that our approach does not align with recommendations for using the monoclonal antibodies aducanumab and lecanemab to treat early AD with proven amyloid-β pathology [18]. However, although the monoclonal antibodies slow the progression of amyloid-related dementias, they require a sophisticated medical infrastructure (i.e., skilled staff, amyloid measurement by PET scan or CSF analysis, MRI machines, infusion centers, and intensive care units for rare, life-threatening side effects) for safe management [18]. These resources are unavailable to most patients at risk despite calls for “equity” in treatment access. Repurposing existing medications is currently the only practical, cost-effective approach.

The major limitation of this retrospective study design is the sampling bias of the estimated effect. The selection of patients was not random: patient age, baseline anxiety, baseline subjective perception of normative cognitive decline, and baseline intellectual curiosity likely influenced the patient’s decision to analyze the ATN profile. Only approximately 5% of the patients at the clinic during the sample time had their plasma ATN profiles determined, implying implicit exclusionary criteria related to younger age, technology skepticism, and perhaps limited cognitive and economic resources to obtain laboratory testing. Another limitation is that the small effect size of the associations between the two biomarkers, pTau181 and NfL, and opioid use, given that r^2^ is approximately 10% of the variance, raises questions about the clinical utility of this plasma biomarker to guide management in isolation. Furthermore, causal inferences regarding the risk vs. benefit of chronic opioid use cannot be derived from a cross-sectional design with nonrandom sampling. The "true" population response to chronic opioid use is very likely highly constrained by psychosocial factors. In Washington state, chronic opioid use is highly stigmatized and regulated with minimal patient access unless a patient has substantial financial resources and geographic proximity to available providers. In addition, providers are hesitant to prescribe opioids due to poorly defined patient characteristics in combination with working in a de facto strict liability regime in case of an overdose death of any type. Only randomized trials or longitudinal single-patient trials will allow for causal inferencing based on clinically relevant effect sizes, a challenging task in today's regulatory environment that is perhaps best addressed by subcutaneous or transdermal buprenorphine treatment as the randomized treatment.

This article integrates two study designs, a retrospective cross-sectional population sampling and two case reports, one of which had longitudinal data, to demonstrate to the practicing clinician that dementia prophylaxis is possible with existing technology and medication. It encourages careful scrutiny of phenotypes suggestive of dementia risk within the practitioner’s clinic and surveillance of the rapidly emerging literature on cost-effective dementia prophylaxis. A passive clinical stance that relies solely on receptive advertisement of branded FDA-approved medications with narrow indications should be reexamined.

The range of future single-patient clinical trials is almost limitless based on the rapidly increasing number of inexpensive generic medications showing efficacy in animal models. One promising study in healthy human volunteers found that one dose of suvorexant acutely decreased the pTau181/Tau181 ratio and the concentration of amyloid-β in the human CSF [18]. Moving forward with single-patient clinical trials, the chief constraint will be gathering experienced advice from statisticians specialized in optimization regarding identifying reliable time constants between dose adjustments or creating a central complex phenotype/endophenotype registry based on single-patient trial data [19].

## Conclusions

We found that plasma pTau181 and NfL were associated with opioid use and that pTau181 could be used to guide medication titration in clinically relevant short-time scales. Single-patient trials in patients with premature neuronal aging, where medication and dosage choices are based on individual ATN profiles, may rapidly improve treatment success. The current study provides a proof of concept; future studies should include more cases with longitudinal measurements of plasma ATN biomarkers for medication optimization.
